# The Relationship between Independent Transfer Skills and Upper Limb Kinetics in Wheelchair Users

**DOI:** 10.1155/2014/984526

**Published:** 2014-08-05

**Authors:** Chung-Ying Tsai, Nathan S. Hogaboom, Michael L. Boninger, Alicia M. Koontz

**Affiliations:** ^1^Human Engineering Research Laboratories, VA Pittsburgh Healthcare System, University of Pittsburgh, Pittsburgh, PA 15206, USA; ^2^Department of Rehabilitation Science and Technology, University of Pittsburgh, Pittsburgh, PA 15260, USA; ^3^Department of Bioengineering, University of Pittsburgh, Pittsburgh, PA 15261, USA; ^4^Department of Physical Medicine and Rehabilitation, University of Pittsburgh, Pittsburgh, PA 15213, USA

## Abstract

Transfers are one of the most physically demanding wheelchair activities. The purpose of this study was to determine if using proper transfer skills as measured by the Transfer Assessment Instrument (TAI) is associated with reduced loading on the upper extremities. Twenty-three wheelchair users performed transfers to a level-height bench while a series of forces plates, load cells, and a motion capture system recorded the biomechanics of their natural transferring techniques. Their transfer skills were simultaneously evaluated by two study clinicians using the TAI. Logistic regression and multiple linear regression models were used to determine the relationships between TAI scores and the kinetic variables on both arms across all joints. The results showed that the TAI measured transfer skills were closely associated with the magnitude and timing of joint moments (*P* < .02, model *R^2^* values ranged from 0.27 to 0.79). Proper completion of the skills which targeted the trailing arm was associated with lower average resultant moments and rates of rise of resultant moments at the trailing shoulder and/or elbow. Some skills involving the leading side had the effect of increasing the magnitude or rate loading on the leading side. Knowledge of the kinetic outcomes associated with each skill may help users to achieve the best load-relieving effects for their upper extremities.

## 1. Introduction 

In 2010 there were about 1.6 million people using wheelchairs for mobility [1]; with that number expanding each year [2]. Wheelchair users must use their upper extremities for almost all activities of daily living (ADLs) such as getting in and out of bed, transferring to a shower or toilet, and transferring in and out of a car [3]. A full-time wheelchair user will perform on average 14 to 18 transfers per day [4]. Transfers are a key element of living an active and productive life and play a vital role in maintaining independence of wheeled mobility device users. If wheelchair users cannot transfer freely, their quality of life and community participation will be severely affected [5].

Transfers are one of the most strenuous wheelchair activities performed [6] and nearly half of wheelchair users do not learn how to use proper transfer techniques during rehab [3]. Incorrect transfer skills may predispose wheelchair users to developing upper limb pain and overuse related injuries, such as rotator cuff tears, elbow pain, and carpal tunnel syndrome [7–11]. The onset of pain can lead to social isolation [5], dependence on others for assistance with ADLs, and increased medical expenditures [7]. Only about half of wheelchair users seek treatment for pain [6, 12, 13] and many feel that their symptoms were not improved after treatment [6, 13, 14]. Therefore, it seems that prevention may be crucial to reducing upper limb pain and overuse injuries. Learning to transfer in a way that reduces forces and awkward joint motions is an important strategy for preserving upper limb function [14].

During transfers, the shoulders often assumed a position of flexion, abduction, and internal rotation [4, 15]. This position brings the glenohumeral head in closer alignment to the undersurface of the acromion and has been identified as a critical risk factor for impinging subacromial soft tissue [16]. Previous studies also indicate that the loading on the upper extremity joints during transfers is greater than any other wheelchair related activity [17]. Transfers have been associated with high peak posterior force and shoulder flexion and adductor moments at the shoulders [17–19]. Large posterior forces at the shoulder are thought to contribute to the development of shoulder posterior instability, capsulitis, and tendinitis [20]. The combination of shoulder posterior and superior forces increases the risk of shoulder impingement syndrome [21]. Furthermore, the elbow has been shown to sustain high superior forces during transfers which may cause nerve compression and result in secondary elbow injuries [19]. Extremes of wrist extension during transfers have also been reported which combined with the weight-bearing loads during transfers may exacerbate wrist injuries such as carpal tunnel syndrome [22, 23]. Using transfer techniques that reduce upper limb joint forces and moments may help prevent injuries [24–27].

The current standard for evaluating transfer technique is observation by the therapist and a qualitative assessment. Transfer technique evaluations are not scientifically oriented and uniform across rehabilitation facilities [3, 28]. Results are impacted by the experience of the therapist and their idea of what constitutes a proper transfer, leading to less precise evaluations and a great degree of variability in transfer skills. The Transfer Assessment Instrument (TAI) is the first tool to standardize the way clinicians evaluate transfer technique and to help identify specific skills to target during transfer training. The items on the TAI were based on clinical practice guidelines [11], current knowledge in the literature [18], and best clinical practices related to transfers. The TAI has acceptable to high inter- and intrarater reliability (intraclass correlation coefficient (ICC) values ranging from 0.72 to 0.88) and good face, content, and construct validity [29–31]. However, no study has associated a clinical assessment of transfer skills to biomechanical changes. The purpose of this study is to examine the relationship between transfer skills as measured with the TAI and upper limb joint loading and to determine if using proper transfer skills as defined by the TAI results in better biomechanical factors that prevent the upper limbs from getting secondary injuries. We hypothesize that better transfer skills (higher scores on the TAI) will correlate with lower magnitudes and rates of rise of forces and moments at the shoulders, elbows, and wrists. Knowledge on the relationship between TAI skills and joint biomechanics will lead to more effective transfer assessments and help to focus training on skills that protect the upper limbs for long term use.

## 2. Methods

### 2.1. Participants

The study was approved by the Department of Veterans Affairs Institutional Review Board. All testing occurred at the Human Engineering Research Laboratories in Pittsburgh, PA. The subjects participating in the study were required to be over 18 years old and one year after injury or diagnosis, use a wheelchair for the majority of mobility (40 hours/per week), and be unable to stand up without support. Individuals with pressure sores within the past year and history of angina or seizures were excluded.

### 2.2. Testing Protocol

**Figure 1:**
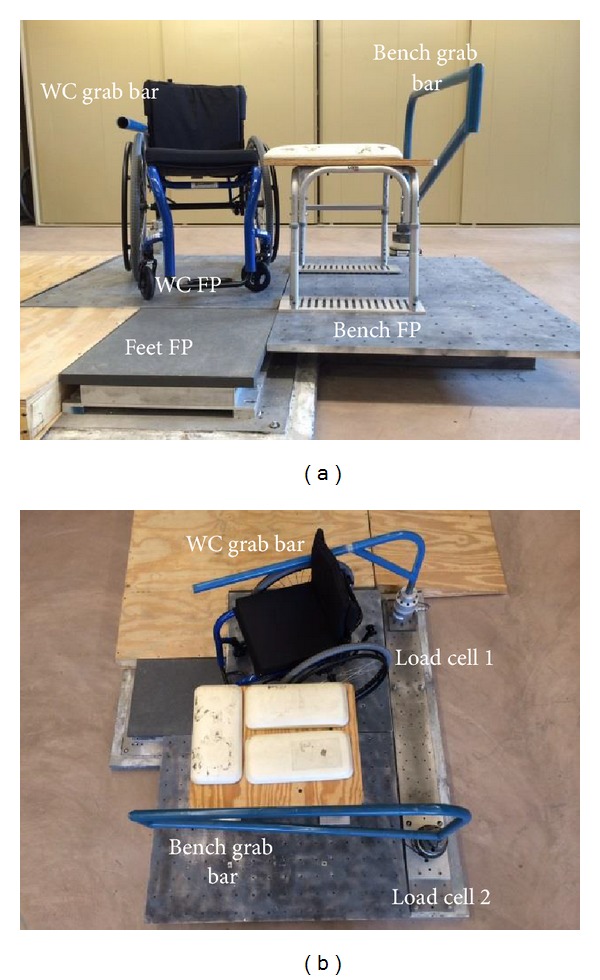
Front (a) and top (b) views of the transfer station. WC: wheelchair; FP: force plate.

**Figure 2:**
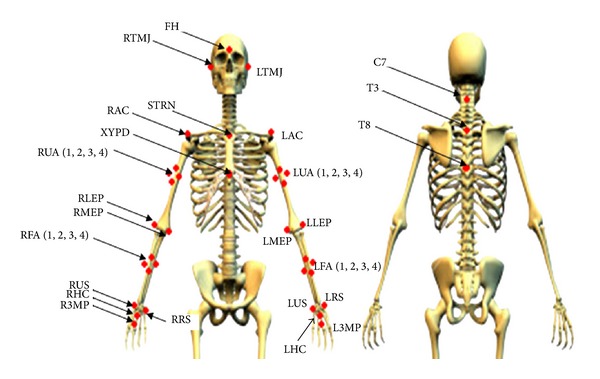
The marker set used in the current study. FH: forehead; RTMJ: right temporomandibular joint; LTMJ: left temporomandibular joint; STRN: sternum; RAC: right acromioclavicular joint; LAC: left acromioclavicular joint; XYPD: xiphoid; RUA: right upper arm; LUA: left upper arm; RLEP: right lateral epicondyle; LLEP: left lateral epicondyle; RMEP: right medial epicondyle; LMEP: left medial epicondyle; RFA: right forearm; LFA: left forearm; RUS: right ulnar styloid; LUS: left ulnar styloid; RRS: right radial styloid; LRS: left radial styloid; RHC: right hand center; LHC: left hand center; R3MCP: right 3rd metacarpophalangeal joint; L3MCP: left 3rd metacarpophalangeal joint; C7: 7th cervical spinous process; T3: 3rd thoracic spinous process; T8: 8th thoracic spinous process.

After written informed consent was obtained, subjects completed a general demographic questionnaire. Anthropometric measures were collected, such as upper arm length and circumference, to determine the center of mass and moment of inertia for each segment [32]. Subjects were asked to position themselves next to a bench, which was at a height level to their own wheelchair seats, on a custom-built transfer station (Figure 1) [33]. The transfer station contains three force plates (Bertec Corporation, Columbus, OH) which were underneath the wheelchair, level bench, and the subject's feet, respectively. Two 6-component load cells (Model MC5 from AMTI, Watertown, MA; Model Omega 160 from ATI, Apex, NC) were attached to two steel beams used to simulate an armrest and grab bar (Figure 1). Subjects were asked to naturally position and secure their wheelchairs in the 3∗3 square foot (91.44 cm by 91.44 cm) aluminum platform that covered the wheelchair force plate. They were also asked to choose where they wanted to position and secure the bench on the other 3∗4 square foot aluminum platform (91.44 cm by 121.92 cm) that covered the bench force plate (Figure 1). The position of the wheelchair grab bar was also adjusted based on the subjects' preference. Reflective markers (Figure 2) were placed on subjects' heads, trunks, and upper extremities to build local coordinate systems [34] for each segment. Marker trajectories were collected at 100 Hz using a ten-camera three-dimensional motion capture system (Vicon, Centennial, CO.) Kinetic data from all the force plates and load cells were collected at 1000 Hz.

Subjects were asked to perform up to five trials of level-height bench transfers. In each trial, subjects needed to perform transfers to and from their own wheelchairs in a natural way. Movement from one surface to the other (e.g., wheelchair to bench) was considered as one transfer. They were provided an opportunity to adjust their wheelchair position and familiarize themselves with the setup prior to data collection. Subjects had time to rest in between trials and additional rest was provided as needed. They were asked to use their own approaches to transferring so their transfer movement pattern and techniques would be as natural as possible. Subjects were asked to place their trailing arm (right arm) on the wheelchair grab bar (Figure 1) when they transferred to the bench on their left side so the reaction forces at the hand could be recorded. On the bench side, subjects were free to place their hand on either the bench or the grab bar. During each trial, up to two study clinicians independently observed and scored each subject's transfer skills using the TAI. All of the participants in the study were evaluated by the same two clinicians. Both were physical therapists who were trained to use the TAI before the study started. The TAI was completed after watching participants perform three to five transfers from the wheelchair to the bench. After independently scoring each subject, the clinicians compared their findings. Any discrepancies in the scoring were discussed and a score reflecting the consensus decision was recorded.

### 2.3. Data Analysis

The biomechanical variables were computed using MATLAB (Mathworks, Inc., Natick, MA, USA). A zero-lag low-pass 4th order Butterworth filter with cutoff frequency of 7 and 5 Hz was used to filter the kinetic and kinematic data, respectively [19]. Only the lift phase of the transfer from the wheelchair to the bench was analyzed in this study. A transfer was determined to begin when a vertical reaction force was detected by the load cell on the wheelchair side grab bar (Figure 1) and ended before a landing spike was detected by the force plate underneath the bench [35]. The end of the lift phase and the beginning of the descent phase are defined by the highest elevated point of the trunk which is indicated by the peak of the C7 and T3 marker trajectories [35]. Hanavan's model was used to calculate center of mass and moment of inertia using the subjects' segment lengths and circumferences [32]. Three-component forces and moments measured by the load cells and the force plates (Figure 1), the marker data of the trunk and upper extremities, and the inertial properties of each body segment were inputs into an inverse dynamic model [36]. Each segment was assumed as a rigid body and linked together by ball and socket joints. The 3rd metacarpophalangeal joint was assumed as the point of force application. The output of the inverse dynamic model included upper extremity net joint forces and moments.

The key kinetic variables included average and maximum resultant forces and moments, and maximum rate of rise of resultant force and moment at the shoulders, elbows, and wrists on both sides. Since shoulder pain is more commonly associated with transfers [7], we analyzed the maximum superior and posterior shoulder forces and extension, abduction, and internal rotation shoulder moments. These variables were selected because they have been linked to shoulder pain, median nerve function, and other upper extremity injuries [18, 21, 24, 25, 37–39]. The resultant force on each joint is indicative of the total joint loading. The maximum rate of rise of resultant force is the peak instantaneous loading rate and impact force on each joint. The resultant moment on each joint represents the rotational demands associated with the muscle forces around the joint and the external forces. The maximum rate of rise of resultant moment indicates the peak rate of moment production on each joint. The superior and posterior shoulder forces were defined as the components of resultant shoulder force acting along the vertical upward and posterior axes of shoulder. Each kinetic variable was normalized by body mass (in kilogram) [17, 18, 40]. Descriptive statistics (means and standard deviations (SD)) were calculated for each variable. Kinetic variables were averaged over a minimum of three and a maximum of five trials. 

The TAI contains two parts—parts 1 and 2. Both parts are scored and averaged to produce a third, final score. Only part 1 item scores and part 1 summary scores were used because the part 1 items evaluate whether the individual used specific transfer skills. Part 2 was not analyzed in this study as it encompasses some of the same transfer skills that are measured in part 1. Part 1 is comprised of 15 items which are scored “yes” (1 point) when the subject performs the specified skill correctly and “no” (0 points) when the subject performs the skill incorrectly or not applicable “(N/A)” which means the item does not apply. The part 1 summary score is the summation of each item's score multiplied by 10, and then divided by the number of applicable items, ranging from 0 to 10 [29]. TAI items that had a 50% response rate or higher in a N/A category or greater than an 80% response rate in the same non-NA category (e.g., yes or no) were not considered for further analysis on the individual item scores. Point-biserial correlations were conducted between the remaining items. Among the items that were highly correlated (*r* > 0.80), one was selected for the logistical modeling analysis (see below).

All of the kinetic data and TAI part 1 summary scores (e.g., continuous variables) were examined for normality using the Shapiro-Wilk test. Point-biserial correlation tests between each TAI item score (e.g., dichotomous variable) and the kinetic variables and Spearman's correlation tests between part 1 summary scores and kinetic variables were conducted to identify relationships with at least a medium effect size (*r* ≥ .30 or ≤−.30 [41]). In order to verify specific kinetic effects of each transfer skill, logistic regression was used to model the association between individual TAI item scores (dichotomous outcome variable) and kinetic variables (predictors). Multiple linear regression was used to model the association between the TAI part 1 summary scores (continuous outcome variable) and kinetic variables (predictors). Separate models were created for the left and right sides. For the logistic regression model, histograms and Q-Q plots were used to check the assumption of no outliers. The assumption of multicollinearity for the kinetic variables (predictors) was tested using the variance inflation factors (VIFs) [42]. The assumption of linear relationships between continuous predictors and the log of the outcome variable was tested by Box-Cox transformation [43]. For the multiple linear regression models, histograms and Q-Q plots were used to check the assumption of no outliers on both predictors and outcome variables. The scatter plot of the standardized residuals against the predicted value was used to test the assumption of linearity. Shapiro-Wilk test was used to check the normality of the error term of the regression model. The assumption of multicollinearity for the predictors was also tested using the VIFs [42]. The assumptions of homoscedasticity and independence for multiple linear regression was checked using the Breusch-Pagan test [44] and Durbin-Watson test [45], respectively.

Backward elimination was used to determine the subset of predictors (kinetic variables) for each TAI outcome variable. The level of significance was set at *P* < .05. All the statistical analyses were performed in SPSS 21 (SPSS Inc., Chicago, IL).

## 3. Results

### 3.1. Participants

Twenty men and three women volunteered to participate in the study.  Table 1 shows summary demographic information. Eighteen subjects had a spinal cord injury (SCI); 14 subjects reported a complete SCI and four subjects an incomplete SCI (three with American Spinal Injury Association (ASIA) Grade B, one with ASIA Grade C). Three subjects had quadriplegia (C4 to C6), 9 had high paraplegia (T2 to T7), and six had low paraplegia (T8 to L3) [46]. The remaining five participants had bilateral tibial and fibular fractures with nerve damage (*n* = 1), double above knee amputation (*n* = 1), muscular dystrophy (*n* = 1), osteogenesis imperfecta (*n* = 1), and myelopathy (*n* = 1).

### 3.2. TAI Variables

Since the TAI part 1 summary scores and final scores were highly correlated (*r* = .97), the TAI part 1 summary scores were used for the multiple regression model. The part 1 summary scores ranged from 3.08 to 10.00 with an average (±SD) of 7.30 (±1.76).  Table 2 shows the items in the part 1 of the TAI. Items 1, 2, 6, 7, 9, and 12 met the inclusion criteria for the logistic models (yes response rate ranges from 39% to 78%, *n* = 23). Items 4, 5, and 15 were not modeled because of the high number of N/A responses. Items 8, 10, 11, 13, and 14 were not modeled because they had too high of a “yes” response rate (e.g., greater than 80% of subjects). Items 3 and 7 scores had the same exact responses for both (*r* = 1). Item 7 scores were modeled because it can be applied to both manual and power wheelchair users, whereas item 3 only applies to manual wheelchair users.

### 3.3. Kinetic Variables

Means and standard deviations of the selected kinetic variables are shown in Table 3.

### 3.4. Correlation Test Results

The TAI part 1 summary and item scores were statistically associated and at least moderately correlated (*r* ≥ .3 or ≤−.3) with one or more of the kinetic variables [41] (Table 4).

### 3.5. Logistic Regression Models for Item Scores

Lower average resultant shoulder force and higher maximum rate of rise of resultant shoulder moment on the leading (left) side were associated with a “yes” score on item 1 (Table 5). Subjects with lower maximum internal rotation shoulder moments on the leading (left) side had an increased likelihood of a “yes” score for item 2. Lower average resultant shoulder moment on the trailing (right) side and lower maximum rate of rise of resultant shoulder moment on the leading (left) side corresponded with a “yes” score on item 6.

On the trailing (right) side, subjects with lower average resultant moment and maximum rate of rise of resultant moment at the elbow were more likely to have a “yes” score on item 7. On the leading (left) side, a higher maximum shoulder extension moment was associated with a “yes” score on item 7.

On the trailing (right) side, a “yes” score on item 9 corresponded with lower average resultant shoulder moment and lower maximum rate of rise of resultant elbow moment. On the leading (left) side, a “yes” score on item 9 was associated with lower maximum rate of rise of resultant shoulder moment, higher maximum internal rotation shoulder moment, lower maximum rate of rise of resultant elbow moment, and higher maximum rate of rise of resultant wrist moment. Subjects with a lower rate of rise of resultant shoulder moment on the leading (left) side were more likely to score a “yes” on item 12.

### 3.6. Multiple Regression Model for Part 1 Score

Lower average resultant trailing (right) elbow moment, lower maximal rate of rise of resultant leading (left) elbow moment, and higher maximal leading (left) shoulder extension moment were associated with proper completion of a greater number of transfer skills overall (higher TAI part 1 score) (Table 6).

## 4. Discussion 

This is the first study to examine the association between proper and improper transfer skills and the resulting forces and moments imparted on the upper limb joints during the transfer process. Specific transfer skills, identified using the TAI, were found to be associated with kinetic variables related to injury risks on the upper extremities [18, 21, 24, 25, 37–39]. Our study sample included a diverse sample of wheelchair users who had a wide range of transfer skills (e.g., part 1 summary scores that ranged from 3.08 to 10.00). Despite differences across studies in measurement techniques and subject characteristics, our kinetic variables were in line with those values reported for level transfers in other studies. For example, the studies from Gagnon and Desroches et al. measured upper limb joint forces and moments during transfers among individuals with SCI and indicated that maximum wrist resultant moment ranged from 0.14 Nm/Kg to 0.48 Nm/Kg and shoulder posterior force on both sides were 2.64 N/kg and 3.14 N/kg, respectively [17, 40].

From the regression model results (Tables 5 and 6), it appears that transfer skills identified by the TAI are closely associated with the magnitude and timing of joint moments. During transfers, the wheelchair user's trunk and his/her arms can be thought as a tripod [47] which forms a closed kinetic chain [48]. The skills used in transfers (e.g., positioning of the wheelchair, using correct handgrips, etc.) cause alterations in the moment arms or the distances separating the hands and trunk center of mass and changes in upper limb joint angles [49] that act along with the external forces to produce the resulting moments. Certain transfer skills helped to reduce the moments imparted on both upper limbs, while other skills had the effects of increasing the magnitudes or rates of loading on the leading (left) arm. Proper completion of the skills related to the trailing (right) arm (part 1 summary score and Items 6, 7 and 9) had the effect of lowering the trailing (right) shoulder and/or elbow peak resultant moment or rate of resultant moment loading. This is significant considering that the trailing arm tends to support a higher percentage of the body weight during sitting-pivot transfers [50, 51].

The six transfer skills as measured by the TAI were modeled because at least 20% of our subject sample scored a “no” for incorrect performance of a particular skill. Four of the six applicable TAI items (transfer skills) dealt with the setup of the wheelchair and body prior to making the transfer. Positioning the wheelchair within 3 inches of the target surface, as measured by item 1, was associated with a reduction in the average resultant shoulder force (*B* = −2.45, *P* = .06) and an increased rate of rise of shoulder resultant moment (*B* = 1.32, *P* = .07) (Table 5) on the leading (left) side. The increase in rate of rise may be associated with a shorter time needed to make the transfer when the body is in a position that is closer to the target surface. A proper angle (20 to 45 degrees) between the wheelchair and transfer surface (item 2) was associated with lower peak internal rotation shoulder moment on the leading (left) side (*B* = −16.53, *P* = .04) (Table 5). Angling the wheelchair next to the target as opposed to parallel parking provides a space that can be used to pivot the trunk and lower body over to the target surface. Angling the wheelchair also allows for the user to clear the rear wheel more easily. The pivoting actions of the trunk and lower body and clearing the pathway to the target surface may have helped to reduce the rotational demands on the leading shoulder.

Proper positioning of the feet (Item 6) can provide wheelchair users with greater dynamic postural control during transfers [18]. About 30% of the body weight during sitting pivot transfers is supported by the feet and legs [51]. Subjects who scored well on this item had lower resultant moments on the trailing (right) shoulder (*B* = −5.73, *P* = .04) and less maximum rate of rise of resultant moment at the leading (left) shoulder (*B* = −1.34, *P* = .06) (Table 5). “Scooting forward” to the front edge of the wheelchair seat before transfers (Item 7) was associated with less trailing (right) elbow moment and its rate of rise (*B* = −13.34 and −3.70, *P* = .09 and .09) (Table 5). Scooting forward brings wheelchair users and their trailing hand positions closer to the target surface which would decrease the lever arm that the applied force is acting through. Our regression model however also indicated that this skill increases leading (left) shoulder extension moment (*B* = 3.91, *P* = .06) (Table 5). The increasing shoulder extension moment may have resulted from a shift in loading from the trailing arm to the leading arm. As mentioned, the trailing arm bears more force in a transfer. Getting closer to the surface allows for placing both hands closer to the trunk center of mass which helps to balance the loading more equally across both arms [52]. For persons who position themselves correctly this will mean seeing less loading on the trailing arm and possibly more loading on the leading arm. In any case higher shoulder extension moment has been shown to increase the risk of pathology, such as ligament edema [24]. Close positioning and appropriate angling wheelchair and foot placement may help to mediate the increased shoulder moments experienced on the leading side.

Item 9 evaluates whether wheelchair users use a correct handgrip on the leading arm within their base of support when performing transfers. Clinical practice guidelines encourage wheelchair users to use handgrips instead of flat hands or fists when performing transfers [11]. Using flat hands during transfers will cause extreme wrist extension which is one factor identified in the etiology of carpal tunnel syndrome, while a closed-finger fist will result in excessive pressure on the metacarpal joints [11, 53]. The use of handgrips can prevent extreme wrist angles, provide more stability, and help apply forces during transfers [11]. During transfers, the handgrip choices are limited by the type of transfer surface and the handgrip option available. For the bench transfer evaluated in this study, subjects could either drape their leading fingers over the edge of the bench with the palm resting on the surface, place a flat palm or fist anywhere on top of the bench, or use the adjacent grab bar. If they used a flat palm, used a closed-finger fist, and/or placed their leading hand outside of where the clinicians felt would be their base of support, the subjects were scored a “no” on this item. Our results from the regression models showed that using a correct leading handgrip (item 9) can lower shoulder resultant moment (*B* = −9.91, *P* = .23) and rate of rise of elbow moment (*B* = −10.38, *P* = .04) on the trailing (right) side and lower the rate of rise of the shoulder and elbow resultant moments on the leading (left) side (*B* = −1.39 and −4.74, *P* = .20 and .14) (Table 5). Because this item combines multiple aspects of handgrips it is difficult to know exactly which attribute (e.g., type of finger grip or hand placement within the base of support) is more responsible for the kinetic outcomes. The rate of rise of the wrist resultant moment increased with better handgrip (*B* = 7.51, *P* = .09) which may be associated with the types of handgrips used by the subjects which were not explicitly documented in this study. Future research should be done to investigate the impact that different types of handgrips used in transfers have on the upper limb joint forces and moments.

Wheelchair users who use the head-hips technique appropriately (Item 12) experienced lesser rate of rise of moment on the leading (left) shoulder (*B* = −.81, *P* = .05) (Table 5). This technique has been associated with an increase in trunk forward flexion and a shift of the trunk center of mass forward and downward to create a moment which can facilitate lifting the buttocks during the transfer [54]. As with setting up the wheelchair angle appropriately, the increased trunk pivot motions may have helped to reduce the rate of rise of resultant shoulder moment.

Wheelchair users with proper overall transfer skills (higher part 1 summary scores) were more likely to experience lower moments on the trailing (right) elbow (*B* = −5.86, *P* < .01) and lower rate of rise of resultant moment on the leading (left) elbow (*B* = −1.13, *P* < .01) but increased extension shoulder moment on the leading (left) side (*B* = 1.94, *P* = .03) (Table 6). Shoulder and elbow movements are related to each other in a close chain activity [48]. As observed with the individual TAI items using good skills can shift loading off of one joint onto another or from one arm to the other. Offloading the elbows and loading the shoulders more may make for a more efficient transfer particularly for individuals who lack elbow extension function. Although triceps muscle function can make a transfer easier (assist with lifting the buttocks off the surface) it is not a primary mover in transfers. The primary movers for transfers are the actions of the pectoralis major muscles, serratus anterior, and latissimus dorsi muscle groups which are all attached to shoulder [17, 55]. The increasing extension shoulder moment may have resulted from the recruitment of the large primary movers, such as the latissimus dorsi and pectoralis major muscles [19, 56] which can drive the movement and shift the body weight during transfers [57].

As noted in our regression models (Tables 5 and 6), some transfer skills as measured by the TAI increase magnitudes and rates of rise of moments. By properly using different transfer skills in tandem, the risks associated with secondary injuries may be minimized. For example, wheelchair users should angle their wheelchairs appropriately relative to the target surface (20–45 degrees) to reduce the large internal rotation shoulder moments on the leading side which can occur when using a proper leading handgrip. Using the head-hip technique (item 12 skill), can reduce the increasing rate of rise of leading shoulder moments which was also associated with close wheelchair positioning. Wheelchair users may need to combine skills to reduce biomechanical loading on the upper extremities, to a greater effect than when utilizing only one or the other. For example, wheelchair users should combine close wheelchair positioning with the scooting forward in their wheelchair to reduce the extension moment on leading shoulder. Taking into consideration the kinetic effects of all transfer skills studied may help to relieve negative effects on the upper extremities during transfers.

## 5. Study Limitations 

The small sample size may have negatively affected the power of the statistical analyses and response rate for some of the TAI items. For example not all of the items could be modeled because subjects were either too proficient on the item or the item did not apply to their transfer. This study only analyzed transfers from a wheelchair to a level-height bench located on the subjects' left side and required them to use the wheelchair side grab bar for positioning of the trailing hand (Figure 1). Subjects were given time to acclimate to the setup prior to testing. Furthermore, a prior study found no differences in muscular demand based on which side (dominant or nondominant) led the transfer or preferred direction of transfer [56]. Wheelchair users have to learn to be flexible with adapting to different setups when they transfer in public places where places to position their hands or the area to position their wheelchairs is limited. Future studies should consider the effects of skills on kinematic variables. Furthermore, the biomechanical effects of transfer training based on TAI principles should be investigated.

## 6. Conclusions

The study shows that the transfer skills that can be measured with the TAI are closely associated with the magnitude and timing of joint moments. Certain transfer skills helped to reduce the moments imparted on both upper limbs, while other skills had the effects of increasing the magnitudes or rates loading on the leading limb. Different skills have different kinetic effects on the upper extremities. Taking into consideration the kinetic effects from all the transfer skills studied may help to reach better load-relieving effects on the upper extremities during transfers. The study provides insight into the impact that a specific skill can have on upper limb loading patterns. As such the TAI may be useful for measuring the effects of a training intervention on reducing upper limb joint loading.

## Figures and Tables

**Table 1:** Participants' demographic information.

Subjects, *n* = 23	Mean ± standard deviation (range)
Age (years)	38.30 ± 11.07 (21–55)
Height (meters)	1.67 ± 0.23 (.99–1.85)
Weight (kilograms)	67.14 ± 19.18 (29.96–98.15)
Average duration of using a wheelchair (years)	13.15 ± 8.13 (1–27.25)

**Table 2:** The items in part 1 of the TAI.

Items in part 1 of the TAI	
(1) The subject's wheelchair is within 3 inches of the object to which he is transferring on to.	
(2) The angle between the subject's wheelchair and the surface to which he is transferring is approximately 20–45 degrees.	
(3) The subject attempts to position his chair to perform the transfer forward of the rear wheel (i.e., subject does not transfer over the rear wheel).	
(4) If possible, the subject removes his armrest or attempts to take it out of the way.	
(5) The subject performs a level or downhill transfer, whenever possible.	
(6) The subject places his feet in a stable position (on the floor if possible) before the transfer.	
(7) The subject scoots to the front edge of the wheelchair seat before he transfers (i.e., moves his buttocks to the front 2/3rds of the seat).	
(8) Hands are in a stable position prior to the start of the transfer.	
(9) A handgrip is utilized correctly by the leading arm (when the handgrip is in the individual's base of support).	
(10) A handgrip is utilized correctly by the trailing arm (when the handgrip is in the individual's base of support).	
(11) Flight is well controlled.	
(12) Head-hip relationship is used.	
(13) The lead arm is correctly positioned. (The arm should not be extremely internally rotated and should be abducted 30–45 deg.)	
(14) The landing phase of the transfer is smooth and well controlled (i.e., hands are not flying off the support surface and the subject is sitting safely on the target surface).	
(15) If an assistant is helping, the assistant supports the subject's arms during the transfer.	

**Table 3:** The mean (±standard deviation (SD)) of the kinetic variables normalized by body mass (kg).

Variables	Trailing (right) side	Leading (left) side
	Mean (±SD)	Mean (±SD)
Shoulder		
AveRF (N/Kg)	2.98 (±0.75)	2.52 (±0.54)
MaxRF (N/Kg)	4.54 (±1.10)	4.24 (±0.97)
MaxRFRate (N/sec∗Kg)	15.95 (±6.09)	13.14 (±5.72)
AveRM (N∗m/Kg)	0.53 (±0.26)	0.60 (±0.17)
MaxRM (N∗m/Kg)	0.87 (±0.38)	1.06 (±0.25)
MaxRMRate (N∗m/sec∗Kg)	3.36 (±1.95)	3.96 (±1.38)
MaxSupF (N/Kg)	1.58 (±0.70)	2.18 (±1.14)
MaxPosF (N/Kg)	3.22 (±1.17)	3.23 (±0.95)
MaxIRM (N∗m/Kg)	0.10 (±0.11)	0.10 (±0.15)
MaxAbdM (N∗m/Kg)	0.43 (±0.21)	0.42 (±0.26)
MaxExtenM (N∗m/Kg)	0.41 (±0.30)	0.70 (±0.32)

Elbow		
AveRF (N/Kg)	2.76 (±0.71)	2.37 (±0.59)
MaxRF (N/Kg)	4.35 (±1.07)	4.20 (±1.03)
MaxRFRate (N/sec∗Kg)	16.06 (±6.00)	4.66 (±2.91)
AveRM (N∗m/Kg)	0.38 (±0.16)	0.21 (±0.10)
MaxRM (N∗m/Kg)	0.62 (±0.23)	0.39 (±0.15)
MaxRMRate (N∗m/sec∗Kg)	2.43 (±1.18)	1.85 (±0.89)

Wrist		
AveRF (N/Kg)	2.69 (±0.70)	2.34 (±0.61)
MaxRF (N/Kg)	4.29 (±1.05)	4.19 (±1.06)
MaxRFRate (N/sec∗Kg)	16.21 (±6.08)	13.17 (±5.74)
AveRM (N∗m/Kg)	0.22 (±0.06)	0.15 (±0.08)
MaxRM (N∗m/Kg)	0.35 (±0.09)	0.26 (±0.14)
MaxRMRate (N∗m/sec∗Kg)	1.34 (±0.57)	0.86 (±0.46)

Ave: average; Max: maximum; RF: resultant force; RFRate: rate of rise of resultant force; RM: resultant moment; RMRate: rate of rise of resultant moment; SupF: superior force; PosF: posterior force; IRM: internal rotation moment; AbdM: abduction moment; ExtenM: extension moment.

**Table 4:** Point-biserial correlation coefficients between TAI items and kinetic variables and Spearman's correlation coefficients between the part 1 summary scores and kinetic variables. The table shows the relationships that were statistically significant and had a medium effect size or larger: *r* ≥ .3 or ≤−.3.

Correlations	Trailing (right) side							Leading (left) side						
	1	2	6	7	9	12	Part 1	1	2	6	7	9	12	Part 1
Shoulder														
AveRF				−.43∗			−.35	−.30	−.31					
MaxRF									−.36					
MaxRFRate			−.31	−.32	−.54∗					−.37				
AveRM	.30		−.52^$^		−.44∗							−.34		
MaxRM	.31		−.47∗		−.49∗									
MaxRMRate	.37		−.51∗		−.52∗			.37		−.55∗		−.39	−.46∗	−.39
MaxSupF														
MaxPosF														
MaxIRM			.37						−.56∗			.42∗		
MaxAbdM						.33			−.32					
MaxExtenM						.31			.35	.30	.43∗			.49∗

Elbow														
AveRF	.30			−.44∗	−.33		−.42							
MaxRF	.31								−.33					
MaxRFRate				−.33	−.57^$^					−.33				−.32
AveRM				−.61^$^	−.49∗		−.39				−.34			
MaxRM				−.59^$^	−.54^$^		−.38				−.32			−.32
MaxRMRate				−.64^$^	−.62^$^		−.40			−.35	−.43∗	−.30	−.36	−.52∗

Wrist														
AveRF				−.44∗	−.34		−.41							
MaxRF					−.30				−.32					
MaxRFRate			−.30	−.32	−.55^$^									
AveRM				−.50^$^			−.49∗	−.31	−.35			.62^$^		
MaxRM				−.38			−.33	−.33	−.36			.64^$^		
MaxRMRate			−.35	−.31	−.46∗							.36		

*P < 0.05; ^$^ *P* < 0.01; Ave: average; Max: maximum; RF: resultant force; RFRate: rate of rise of resultant force; RM: resultant moment; RMRate: rate of rise of resultant moment; SupF: superior force; PosF: posterior fore; IRM: internal rotation moment; AbdM: abduction moment; ExtenM: extension moment.

**Table 5:** Logistic regression model results for each TAI item. Odds ratio (Exp(*B*)) is shown for the predictors that significantly contributed to predicting the TAI item scores. The Negelkerke *R* ^2^ value for each model is reported.

Item	Variables	*B*	*χ* ^2^	Sig.	Exp(*B*)	Model results
Item 1: the subject's wheelchair is within 3 inches of the object to which he is transferring on to.	Leading (left) shoulder AveRF	−2.45	3.55	.06		*χ* ^ 2^(2, *N* = 23) = 8.72, *P* = .01, *R* ^2^ = .42
	Leading (left) shoulder MaxRMRate	1.32	3.39	.07		

Item 2: the angle between the subject's wheelchair and the surface to which he is transferring to is approximately 20–45 degrees.	Leading (left) shoulder MaxIRM∗	−16.53	4.29	.04	.00	*χ* ^ 2^(1, *N* = 23) = 9.09, *P* < .01, *R* ^2^ = .46

Item 6: the subject places his feet in a stable position (on the floor if possible) before the transfer.	Leading (left) shoulder MaxRMRate	−1.34	3.67	.06		*χ* ^ 2^(1, *N* = 23) = 7.86, *P* < .01, *R* ^2^ = .42
	Trailing (right) shoulder AveRM∗	−5.73	4.19	.04	.00	*χ* ^ 2^(1, *N* = 23) = 6.76, *P* < .01, R^2^ = .37

Item 7: the subject scoots to the front edge of the wheelchair seat before he transfers (i.e., moves his buttocks to the front 2/3rds of the seat).	Leading (left) shoulder MaxExtenM	3.91	3.54	.06		*χ* ^ 2^(1, *N* = 23) = 4.70, *P* = .03, *R* ^2^ = .27
	Trailing (right) elbow AveRM	−13.34	2.96	.09		*χ* ^ 2^(2, *N* = 23) = 14.78, *P* < .01, *R* ^2^ = .69
	Trailing (right) elbow MaxRMRate	−3.70	2.82	.09		

Item 9: a handgrip is utilized correctly by the leading arm (when the handgrip is in the individual's base of support).	Leading (left) shoulder MaxRMRate	−1.39	1.62	.20		*χ* ^ 2^(4, *N* = 23) = 18.29, *P* < .01, *R* ^2^ = .74
	Leading (left) shoulder MaxIRM	22.10	1.85	.17		
	Leading (left) elbow MaxRMRate	−4.74	2.21	.14		
	Leading (left) wrist MaxRMRate	7.51	2.83	.09		
	Trailing (right) shoulder AveRM	−9.91	1.43	.23		*χ* ^ 2^(2, *N* = 23) = 19.92, *P* < .01, *R* ^2^ = .79
	Trailing (right) elbow MaxRMRate∗	−10.38	4.07	.04	.00	

Item 12: Head-hip relationship is used.	Leading (left) shoulder MaxRMRate	−.81	3.82	.05		*χ* ^ 2^(1, *N* = 23) = 5.13, *P* = .02, *R* ^2^ = .27

Note: *the predictor significantly contributed to the regression model. *B*: unstandardized regression coefficients; Sig.: significance; Exp(*B*): odds ratio; Ave: average; Max: maximum; RMRate: rate of rise of resultant moment; RF: resultant force; IRM: internal rotation moment; RM: resultant moment; ExtenM: extension moment.

**Table 6:** Multiple linear regression analysis summary for predicting part 1 score.

Variable	*B*	SEB	*β*	sr^2^	Sig.	Regression model
Trailing (right) elbow AveRM∗	−5.86	2.02	−.53	.29	<.01	*F*(1,21) = 8.40, *P* < .01, *R* ^2^ = .29

Leading (left) shoulder MaxExtenM	1.94	.85	.35	.12	.03	*F*(2,20) = 12.54, *P* < .01, *R* ^2^ = .56
Leading (left) elbow MaxRMRate∗	−1.13	.30	−.57	.31	<.01	

Note: *the predictor significantly contributed to the regression model. *B*: unstandardized regression coefficients; SEB: standard error of the unstandardized regression coefficients; *β*: standardized regression coefficients; sr^2^: squared semipartial correlations; Sig.: significance; Ave: average; Max: maximum; RM: resultant moment; ExtenM: extension moment; RMRate: rate of rise of resultant moment.
